# Change in Air Quality during 2014–2021 in Jinan City in China and Its Influencing Factors

**DOI:** 10.3390/toxics11030210

**Published:** 2023-02-24

**Authors:** Qingchun Guo, Zhenfang He, Zhaosheng Wang

**Affiliations:** 1 School of Geography and Environment, Liaocheng University, Liaocheng 252000, China; 2 Institute of Huanghe Studies, Liaocheng University, Liaocheng 252000, China; 3 State Key Laboratory of Loess and Quaternary Geology, Institute of Earth Environment, Chinese Academy of Sciences, Xi’an 710061, China; 4 State Key Laboratory of Urban and Regional Ecology, Research Center for Eco-Environmental Sciences, Chinese Academy of Sciences, Beijing 100085, China; 5 National Ecosystem Science Data Center, Key Laboratory of Ecosystem Network Observation and Modeling, Institute of Geographic Sciences and Natural Resources Research, Chinese Academy of Sciences, Beijing 100101, China

**Keywords:** air quality, influencing factors, PM_2.5_, PM_10_, COVID-19

## Abstract

Air pollution affects climate change, food production, traffic safety, and human health. In this paper, we analyze the changes in air quality index (AQI) and concentrations of six air pollutants in Jinan during 2014–2021. The results indicate that the annual average concentrations of PM_10_, PM_2.5_, NO_2_, SO_2_, CO, and O_3_ and AQI values all declined year after year during 2014–2021. Compared with 2014, AQI in Jinan City fell by 27.3% in 2021. Air quality in the four seasons of 2021 was obviously better than that in 2014. PM_2.5_ concentration was the highest in winter and PM_2.5_ concentration was the lowest in summer, while it was the opposite for O_3_ concentration. AQI in Jinan during the COVID epoch in 2020 was remarkably lower compared with that during the same epoch in 2021. Nevertheless, air quality during the post-COVID epoch in 2020 conspicuously deteriorated compared with that in 2021. Socioeconomic elements were the main reasons for the changes in air quality. AQI in Jinan was majorly influenced by energy consumption per 10,000-yuan GDP (ECPGDP), SO_2_ emissions (SDE), NOx emissions (NOE), particulate emissions (PE), PM_2.5_, and PM_10_. Clean policies in Jinan City played a key role in improving air quality. Unfavorable meteorological conditions led to heavy pollution weather in the winter. These results could provide a scientific reference for the control of air pollution in Jinan City.

## 1. Introduction

Air pollution affects food production, climate change, ecosystem health, traffic safety, the spread of SARS-CoV-2 (COVID-19 virus) and other viruses, human health, and socio-economic development [1,2,3,4,5,6,7]. Air pollution is a hazardous element for respiratory tract infections that carries microorganisms and affects the immunity of the body. There is an obvious positive correlation between atmospheric pollution and newly confirmed cases of COVID-19 [8]. Fine particulate matter (PM_2.5_) causes about 8.9 million premature deaths globally per year [9]. The risk of premature death attributable to PM_2.5_ rose from about 1.7 million in 2002 to about 2.1 million in 2017 in China [10]. During 2011–2015, about 209,000 people died in Jinan City. The increase in air pollution in Jinan City led to an increase in non-accidental mortality [11]. Clean air policies could decrease the effect of air pollution on well-being [12].

Since 2013, China implemented an Air Clean Plan. China’s air quality has undergone significant changes. From January to March 2013, China experienced extremely serious and continuous haze pollution [13]. Owing to strict emission controls, PM_2.5_ concentration in China fell by about 30–50% from 2013 to 2018 [14]. The concentrations of PM_2.5_, SO_2_, PM_10_, and CO reduced every year during the period 2015–2017 [15]. The Clean Air Plan from 2018 to 2020 has led to a decrease in NO_2_ by about 15.7%, but the COVID-19 lockdown steps have resulted in an about 27% decrease [16].

The COVID-19 pandemic has had a significant influence on air quality as a result of changes in human behavior [17]. As of 2 November, 2022, 628,035,553 confirmed cases and 6,572,800 deaths have been reported globally (World Health Organization (WHO)). In order to curb the COVID-19 pandemic, a great many countries took dramatic measures to reduce interpersonal interaction, including strict isolation, prohibition of public gatherings, restriction of public transportation, encouragement to keep social distance, curfews, and even lockdown of entire cities. City lockdowns resulted in an emphatic improvement in air quality in China [18]. The air quality in Liaocheng during the COVID epoch in 2020 was apparently better than those in the same epochs in 2019 [19]. The air quality during the COVID epoch in 2020 was evidently better than that in 2021 in the Beijing–Tianjin–Tangshan (BTT) area [20]. NO_2_ concentrations during the COVID-19 lockdown period decreased slightly by 8.2% over the urban ambient station in the Metro of Atlanta, USA [21]. PM_2.5_ concentrations in Hanoi, Vietnam, were about 14–18% lower during the COVID-19 epoch than before this era, but CO concentrations had a significant decline by about 28–41% [22]. The control measures during the COVID-19 pandemic reduced NO_2_ levels in China [23]. PM_2.5_ concentrations, during the COVID-19 lockdown in 2020, was significantly reduced compared to the period before the lockdown in Shanghai [24].

Serious air pollution events are usually affected by local pollutant emissions and meteorological conditions. Although small in scale, they are also affected by climate change on a broad time scale [25]. From 2013 to 2019, the increase in O_3_ pollution in China was jointly affected by human factors and meteorological effects [26]. Meteorological factors mainly include high temperatures and atmospheric circulation, while human factors mainly refer to the massive emission of O_3_ precursors, such as NOx and VOCs [26]. The increasing trend of O_3_ concentration in China is chiefly attributable to the trend of meteorological factors, for instance, solar radiation and air temperature. However, the trend of PM_2.5_ concentration is mainly caused by emission reduction of PM_2.5_ and its precursors (CO, NO_2_, SO_2_, and formaldehyde). In addition, relative humidity is positively correlated with PM_2.5_ concentration [27]. PM_2.5_ concentration has the strongest correlation with relative humidity, temperature, and atmospheric pressure in China [28]. Humidity and wind are the key drivers of the extreme value of PM_2.5_ in China [29]. The numerical model experiments show that a higher temperature, higher relative humidity, and breeze favor the increase in PM_2.5_ level, while the growth of O_3_ concentration is primarily attributable to far hotter and drier meteorological conditions [30,31].

China’s socio-economic factors mainly include the number of cars, energy consumption, the ratio of the secondary industry in GDP, GDP per capita (GDPPC), green coverage, and scientific and technological expenditure. In the regional level, the socio-economic factors of Shandong Peninsula and Beijing–Tianjin–Hebei are different [32]. Government technology expenditure, GDPPC, and population density all have remarkable negative overall impacts on AQI values in the Yellow River Economic Belt of China. Nevertheless, green coverage and secondary industry ratio have noticeable positive total effects [33]. GDPPC is negatively correlated with PM_2.5_ concentration in 30 OECD countries. Expansion of service industry reduces PM_2.5_ concentrations [34]. In short, socio-economic factors have a two-way impact on air pollution. The air quality at different time scales and its driving factors are still unclear.

Few low-cost monitors for air quality are accurately tested. A stable, easy to use, and reproducible platform was developed. In these laboratory conditions, the comparison between the low-cost sensors and calculated concentration was shown to be linear. A complete validation of a low-cost sensor was achieved by its application in a real indoor place. Good correlation between the reference methods and uHoo measurements of PM_2.5_ and O_3_ concentrations was achieved [35]. Two methods are proposed to show results of field measurements and urban climate simulations using the ENVI-met software suite. Based on the measured microclimate data and comfort survey conducted in downtown Curitiba, Brazil, the influence of street geometry on ambient temperatures and daytime pedestrian comfort levels was evaluated, using the sky-view factor (SVF) as an indicator of the complexity of the urban geometry. The influence of street orientation relative to prevailing winds and the resulting effects of ventilation (air speed and spatial distribution) on the dispersion of traffic-generated air pollutants were additionally analyzed through computer simulations. Results show the influence of urban geometry on human thermal comfort in pedestrian streets and on the outcomes of pollutant dispersion scenarios [36].

Urban air quality can have serious impacts on people who use indoor and outdoor spaces. Five classrooms equipped with air conditioners or ceiling fans in Hong Kong (HK) were selected for investigation of indoor and outdoor air quality. CO_2_, SO_2_, NO, NO_2_, PM_10_, and formaldehyde (HCHO) concentrations and total bacteria counts were monitored in both indoor and outdoor conditions. The average respirable PM concentrations were higher than the Hong Kong target, and the maximum indoor PM_10_ level exceeded 1000 mg/m^3^. Indoor CO_2_ concentrations usually exceeded 1000 mL/L in air-conditioning and ceiling fan classrooms, indicating inadequate ventilation. Maximum indoor CO_2_ level reached 5900 mL/L during class at the classroom with cooling tower ventilation. Other pollution parameters complied with the standards. The two most important classroom air quality problems in Hong Kong were PM_10_ and CO_2_ levels [37]. Simultaneous measurements of outdoor and indoor pollution were performed at three schools in Lisbon. VOCs, formaldehyde, and NO_2_ concentrations were passively monitored for two weeks. Bacterial and fungal colony-forming units and comfort parameters were also monitored at classrooms and playgrounds. The highest indoor levels of NO_2_ (40.3 µg/m^3^), CO_2_ (2666 µg/m^3^), VOCs (10.3 µg/m^3^), formaldehyde (1.03 µg/m^3^), and bioaerosols (1634 CFU/m^3^) and some indoor/outdoor ratios greater than unity indicate that indoor sources and building conditions might have negative effects on the air indoors. Increasing ventilation rates and use of low-emission materials would contribute towards improving indoor air quality [38].

Jinan, also known as the spring city, is the capital city of Shandong Province. It borders Mount Tai in the south and spans the Yellow River in the north, with a total area of 10,244.45 square kilometers. In 2021, Jinan had a permanent population of 9,336,000 and a GDP of 1143.222 billion yuan. It belongs to the monsoon climate, with an annual atmospheric temperature of 14.2 °C and an annual precipitation of 548.7 mm. In 2013, the Jinan Government announced the Jinan air pollution prevention action plan. In 2014, Jinan implemented the “Ten Actions” to further strengthen air pollution prevention. In 2018, the Jinan Government issued the program to win the blue-sky defense battle.

In the present research, the air pollution in Jinan City, a highly polluted city, including six air pollutant concentrations and AQIs from 2014 to 2021 are studied. The temporal resolution (e.g., annual, seasonal, monthly, and hourly) is discussed. It will be extremely helpful to investigate such highly resolved data to evaluate the trends in hourly air quality and thus provide insights on air pollutant formation.

## 2. Material and Methods

The air pollution during 2014–2021 in Jinan City in China was investigated. The air quality index (AQI) and six air pollutant concentrations in Jinan City were analyzed (http://www.aqistudy.cn/) (accessed on 3 December 2022). Starting on 23 January 2020, the Chinese government implemented different levels of lockdown restrictions in different cities in order to slow down the transmission of COVID-19. Jinan implemented lockdown restrictions since 1 February 2020, and lifted it since 1 May 2020. These datasets from 2020 to 2021 were separated into two sections: epoch I (1 February to 30 April); epoch II (1 May to 31 December). Socio- economic data were obtained from the Statistics Bureau of Jinan, including GDPPC, population (PO), and ECPGDP. The air pollution emissions were obtained from the Statistics Bureau of Jinan, including SDE, NOE, and PE. The meteorological data were obtained from the Meteorological Bureau of Jinan, including maximum wind speed (MWS), minimum air pressure (MAP), precipitation (PR), average air temperature (AAT), sunshine hours (SH), and average relative humidity (ARH). The AQI could quantitatively express air pollution. AQI was obtained from ‘Technical Regulation on Ambient Air Quality Index (on trial)’ (HJ 633–2012). The six ranks are displayed in Table 1. Daily individual AQI (IAQI) is calculated from the concentrations of individual pollutants (six air pollutants), and the AQI value is determined to be the maximum IAQI of the six pollutants.

AQI and IAQI are calculated from the following equations:

(1) $$\mathrm{AQI}=\max({\mathrm{IAQI}}_{1}{,\mathrm{IAQI}}_{2}{,L,\mathrm{IAQI}}_{m})$$

where IAQI is the individual AQI and m is the pollutant, and

(2) $${\mathrm{IAQI}}_{m}=\frac{{\mathrm{IAQI}}_{h}-{\mathrm{IAQI}}_{l}}{{\mathrm{BP}}_{h}-{\mathrm{BP}}_{l}}{(C}_{m}-{\mathrm{BP}}_{l}{)+\mathrm{IAQI}}_{l}$$

where IAQI_m_ is the individual AQI of pollutant m, C_m_ is the concentration of pollutant m, BP_h_ is the high-value pollutant concentration limit, BP_l_ is the low-value pollutant concentration limit, IAQI_h_ is the individual AQI corresponding to BP_h_, and IAQI_l_ is the individual AQI corresponding to BP_l_. There are two values in the air quality standard: primary standard and secondary standard.

Pearson correlation coefficient (*R*) is used to explore the influence of socioeconomic and meteorological factors on air quality, and analyze the relationship between air pollutants [17]. *R* is calculated by the following equations:

(3) $$R=\frac{\sum \left(E_{p}-\overset{-}{E})(F_{p}-\overset{-}{F}\right)}{\sqrt{\sum {\left(E_{p}-E\right)}^{2}{\left(F_{p}-\overset{-}{F}\right)}^{2}}}$$

$E_{p}$ denotes the air pollutants, $F_{p}$ denotes the influence factors, $\overset{-}{E}$ is the mean of the air pollutants, and $\overset{-}{F}$ is the mean of the influence factors.

## 3. Results and Discussion

In the present research, firstly, changes in air quality in Jinan City during 2014–2021 were analyzed. Secondly, the causes of air quality changes were clarified. Thirdly, changes in air quality between the COVID and the post-COVID epoch in Jinan City were analyzed and discussed. At last, seasonal and monthly variations of air quality were analyzed.

### 3.1. Interannual Changes in Air Quality in Jinan City

**Figure 1:**
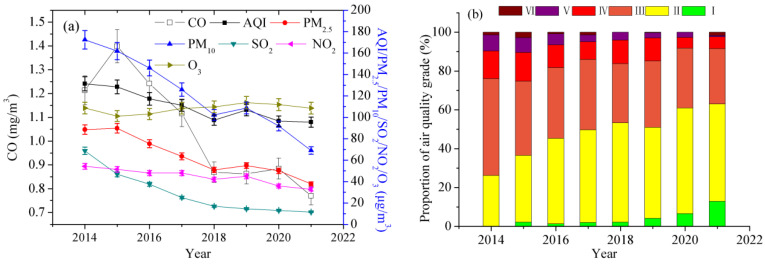
Interannual variations in air quality in Jinan City during 2014–2021.(**a**) the annual average concentrations of PM_10_, PM_2.5_, NO_2_, SO_2_, CO, and O_3_ and AQI values; (**b**) the proportions of AQI ranks.

The annual average concentrations of air pollutants decreased significantly from 2014 to 2021 (Figure 1a). Consequently, the values in 2021 were 27.3%, 57.3%, 60.0%, 83.3%, 39.5%, 36.6%, and 0.1%, which were respectively lower than those in 2014. Air quality in 2021 was conspicuously better than that in 2014. However, the annual mean concentrations of PM_2.5_, NO_2_, and PM_10_ in Jinan City during the period 2014–2021 far exceeded the annual mean guideline of WHO. The trend in O_3_ concentrations is eye-catching. The O_3_ concentrations were approximately constant and thus did not show a substantial improvement unlike other pollutants. This is because the emission of VOCs in Jinan show little change.

We estimate the AQI ranks in Jinan City during 2014–2021 (Figure 1b). From 2014 to 2021, the total ratios of rank I and rank II increased from 26.2% to 63.0%, but the total ratios of ranks IV–VI declined from 24.0% to 8.5%, which illustrates that the air quality was extremely upgraded.

### 3.2. The Association between the Air Quality in Jinan City and Influencing Factors

The changes in air quality are chiefly affected by natural conditions and socio-economic conditions. With the implementation of the policies in Jinan, obvious declines in the six air pollutant concentrations and AQI values emerged from 2014 to 2021.

The related coefficients (R) between air quality, meteorological factors, and socio-economic factors were comparatively good (Table 2). On the annual time scale, from 2014 to 2021, the sample size (N) was eight. Air quality (AQI, PM_10_, SO_2_, PM_2.5_, CO, and NO_2_ concentrations) was positively correlated with MAP, AAT, ARH, and SH and was negatively correlated with maximum wind speed (MWS) and precipitation (PR). However, except for precipitation (PR), the correlations between O_3_ concentration and meteorological factors were the opposite of those of air quality. High temperature, long sunshine, low humidity, low cloud cover, and low wind speed are conducive to ozone generation. However, low pressure and high humidity are conducive to the formation of PM_2.5_. Meteorological factors have a conspicuous influence on the change in air quality. Air pollution index (API) in Xi’an and Lanzhou was strongly related to average temperature, minimum temperature, and maximum temperature [39]. PM_10_, SO_2_, PM_2.5_, and CO concentrations are mainly affected by dew point temperature and air pressure, but O_3_ and NO_2_ concentrations are mainly affected by air temperature and boundary layer height, respectively [40].

On the daily time scale, from 2014 to 2021, the sample size (N) was 2922. Air quality (AQI, PM_10_, SO_2_, PM_2.5_, CO, and NO_2_ concentrations) was positively correlated with MAP and negatively correlated with AAT, maximum wind speed (MWS), and precipitation (PR). However, except for precipitation (PR), the correlations between O_3_ concentration and meteorological factors were the opposite of those of air quality. ARH was positively correlated with AQI and PM_2.5_ and CO concentrations and was negatively correlated with PM_10_, SO_2_, O_3_, and NO_2_ concentrations. SH was positively correlated with PM_10_, SO_2_, NO_2_, and O_3_ concentrations and was negatively correlated with AQI and PM_2.5_ and CO concentrations.

Air pollutant emissions affect air quality. Air quality was positively correlated with SDE, NOE, and PE. The reduction of NOx from 2013 to 2017 helped to control the total production of O_3_ in China [41].

Studying the relationship between the socio-economic system and air quality will help China achieve the goal of sustainable development. Air quality was positively correlated with ECPGDP and negatively correlated with GDPPC and PO. The correlations between AQI and ECPGDP were the best (R = 0.943). From 2014 to 2020, cleaner production and energy consumption control contributed to the largest reduction of PM_2.5_ concentration in China [42]. The impact of GDPPC on haze pollution confirms the relationship of environmental Kuznets curve (EKC) [43].

Social economy also affects the emission of air pollutants. GDP was correlated well with annual emissions of ambient species (PM_2.5_, PM_10_, and SO_2_). GDP per capita correlated with annual emissions of ambient species (PM_2.5_, PM_10_, and SO_2_) in 11 cities around the Bohai Sea. For most cities, the emission and energy use per GDP decreased with the enhancements of economic growth, following the environmental Kuznets curves [44].

The correlations between the six air pollutant concentrations and AQI values are shown in Table 3.

On the annual time scale, from 2014 to 2021, the sample size (N) was eight. The correlation (R) between AQI and PM_2.5_ concentration was the best (R = 0.977), followed by PM_10_ concentration (R = 0.970). These results indicated that PM_2.5_ and PM_10_ concentrations were the major factors affecting AQI. The correlation between PM_10_ and PM_2.5_ concentrations was the best (R = 0.977) and the ratio of PM_2.5_ to PM_10_ was 52.3%, indicating that PM_2.5_ was a large proportion of PM_10_. The strong relationship between PM_2.5_ and NO_2_ concentrations (R = 0.923) implies that NO_2_ plays a significant effect in the formation of PM_2.5_. However, the correlations between O_3_ and PM_10_, SO_2_, PM_2.5_, NO_2_, and CO concentrations were negative. The good relationship between O_3_ and NO_2_ concentrations (R = −0.455) suggested that NO_2_ is an important factor in the formation of O_3_. Therefore, NO_2_ plays a very important effect in the formation of PM_2.5_ and O_3_. The weak negative relationship between O_3_ and PM_2.5_ concentrations indicates the complex interaction between O_3_ and PM_2.5_, and the reasons behind it need to be further studied. The association between PM_2.5_ and O_3_ concentrations is influenced by the atmospheric oxidizing capacity magnitude [45].

On the daily time scale, from 2014 to 2021, the sample size (N) was 2922. The correlation (R) between AQI and PM_10_ concentration was the best (R = 0.768), followed by PM_2.5_ concentration (R = 0.765). The correlation between PM_10_ and PM_2.5_ concentrations was the best (R = 0.856). Nevertheless, the correlations between O_3_ and PM_10_, SO_2_, PM_2.5_, NO_2_, and CO concentrations were negative. The trend of the correlations each year was similar to those from 2014 to 2021. PM always was the major factor affecting AQI each year.

Atmospheric oxidation capacity (AOC) refers to the oxidation capacity of atmospheric chemical processes in primary pollutants, generally expressed by the concentration of oxidants. The main atmospheric oxidants are HO_2_, OH, and NO_3_ free radicals. AOC is closely related to the generation of secondary pollutants. In recent years, the decrease in PM_2.5_ concentration and the increase in O_3_ concentration in China were caused by the increase in AOC. In particular, OH free radicals can react with VOCs to generate peroxy radicals (such as HO_2_), which continue to react with NO to generate NO_2_ and participate in the generation of O_3_ after photolysis, leading to the increase in O_3_ concentration. On the other hand, the concentration of free radicals such as OH increases, which increases the oxidation rate of SO_2_, NOx, and VOCs and accelerates the gas phase formation of sulfate and nitrate [46].

As an important oxidant, O_3_ can affect the generation of sulfate, nitrate, ammonium salt, and secondary organic aerosol in PM_2.5_. The reduction of PM_2.5_ concentration leads to an increase in ozone. PM_2.5_ inhibits the secondary chemical formation of ozone through the heterogeneous absorption of HO_2_ free radicals and NOx. The inhibition of PM_2.5_ on ozone will also cause ozone generation to be more affected by VOC emissions, that is, the sensitivity of ozone to NOx emission reduction will be reduced [47].

### 3.3. Seasonal Changes in Air Quality in Jinan City during 2014–2021

**Figure 2:**
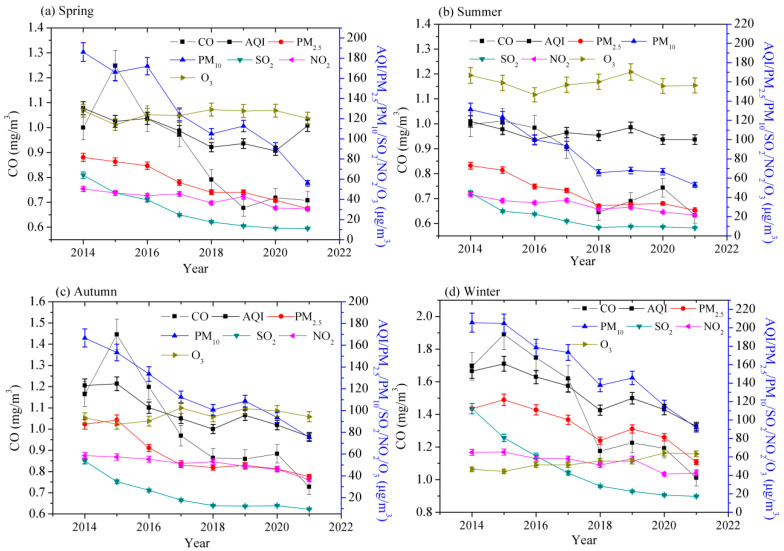
Seasonal variations in air quality in Jinan City during 2014–2021. (**a**) the changes of air quality in spring; (**b**) the changes of air quality in summer; (**c**) the changes of air quality in autumn; (**d**) the changes of air quality in winter.

The air quality in Jinan also has significant characteristics due to seasonal variations. As shown in Figure 2, the seasonal average concentrations of PM_10_, SO_2_, PM_2.5_, NO_2_, and CO were the lowest in summer and were the highest in winter, while the trends of O_3_ concentration and the other five pollutants were obviously different, the highest being in summer and the lowest in winter. Air quality in the four seasons of 2021 was obviously better than that in 2014. Because of heating in winter, the air pollutant emissions in winter were apparently higher than that in other three seasons, which is the fundamental reason for the frequent appearance of serious pollution in Jinan in winter. The high concentrations of NOx and VOCs in the atmosphere, resulting in enhanced atmospheric oxidation, are the critical elements for fast growth of secondary PM_2.5_ with heavy pollution in winter. Unfavorable meteorological conditions cause a prominent reduction in regional environmental capacity, which is a necessary condition for the formation of heavily polluted weather in winter. Regional transmission has a conspicuous effect on PM_2.5_ concentration in winter. In contrast to the negative impact on PM_2.5_ concentration in summer, higher humidity is conducive to the formation of PM_2.5_ in winter because the hygroscopicity of particles increases [48]. In summer, the temperature often rises to more than 32 °C and the sunlight is sufficient, leading to more intense VOC emissions from biological sources [49]. Higher temperature can improve the formation of O_3_ by accelerating the photochemical reaction rates and boosting the biological emission of VOCs [50]. Therefore, air temperature and sunshine play a leading role in the O_3_ concentration in Jinan in summer.

As could be seen from Figure 2a, the values in Jinan City in spring of 2021 were 13.3%, 62.0%, 69.9%, 82.8%, 39.7%, 29.2%, and 6.0% respectively lower than those in 2014. Air quality in Jinan City in spring of 2021 was obviously better than that in 2014. Similarly, for summer (Figure 2b), the values in 2021 were 16.0%, 63.5%, 59.6%, 81.3%, 49.6%, 36.6%, and 6.2% respectively lower than those in 2014. Air quality in summer of 2021 was obviously better than that in 2014. For autumn (Figure 2c), the values in 2021 were 38.2%, 55.0%, 54.8%, 82.6%, 37.5%, 37.5%, and −1.1% respectively lower than those in 2014. Air quality in Jinan City in autumn of 2021 was remarkably better than that in 2014. For winter (Figure 2d), the values in 2021 were 39.2%, 51.6%, 55.4%, 84.6%, 34.4%, 40.3%, and −35.7% respectively lower than those in 2014. Air quality in winter of 2021 was remarkably better than that in 2014. In short, the air quality in Jinan City in 2021 was better than that in 2014 in the four seasons.

Table 4 displays the changes of the ratios of air quality ranks in the four seasons from 2014 to 2021. In spring, the ratios of ranks I and Ⅱ increased by 4.4% and 34.9% from 2014 to 2021, respectively. Similarly, in summer, I and Ⅱ increased by a corresponding 13% and 9.6%, respectively. In autumn, I and Ⅱ increased by a corresponding 27.5% and 12.1%, respectively. In winter, I and Ⅱ increased by a corresponding 6.7% and 39.1%, respectively. The ratios of rank I increase the most in autumn, and the ratios of rank Ⅱ increase the most in winter. In the four seasons, the air quality in 2021 was much better than that in 2014.

### 3.4. Comparison of Air Quality between the COVID Epoch in 2020 and the Same Epoch in 2021 in Jinan City

The mean daily six air pollutant and AQI values in Jinan City from 1 February to 30 April, during 2020–2021, are displayed in Table 5. The values were 25.0%, −18.8%, −24.9%, 8.6%, 6.5%, −1.9%, and −2.2% higher in 2021 than in 2020. These outcomes explain that the AQI in Jinan City during the COVID epoch in 2020 was noteworthily lower compared with that during the same epoch in 2021. During the COVID epidemic in 2020, control measures such as staying at home, closing factories, and reducing traffic played a key role in improving AQI in Jinan City.

### 3.5. Changes in Air Quality during the Post-COVID Epoch in Jinan City

The mean daily six air pollutant concentrations and AQI values in Jinan City from May 1 to December 31, during 2020–2021, are displayed in Table 6. The values were 4.2%, 10.9%, 17.4%, 12.5%, 10.3%, 10.7%, and 10.1% lower in 2021 than during the post-COVID epoch in 2020. These outcomes imply that the air quality in Jinan City during the post-COVID epoch in 2020 noticeably deteriorated compared with that during the same epoch in 2021. Clean policies in Jinan City played a key role in improving air quality in 2021.

### 3.6. Monthly Changes in Air Quality in Jinan City from the COVID Epoch to the Post-COVID Epoch

**Figure 3:**
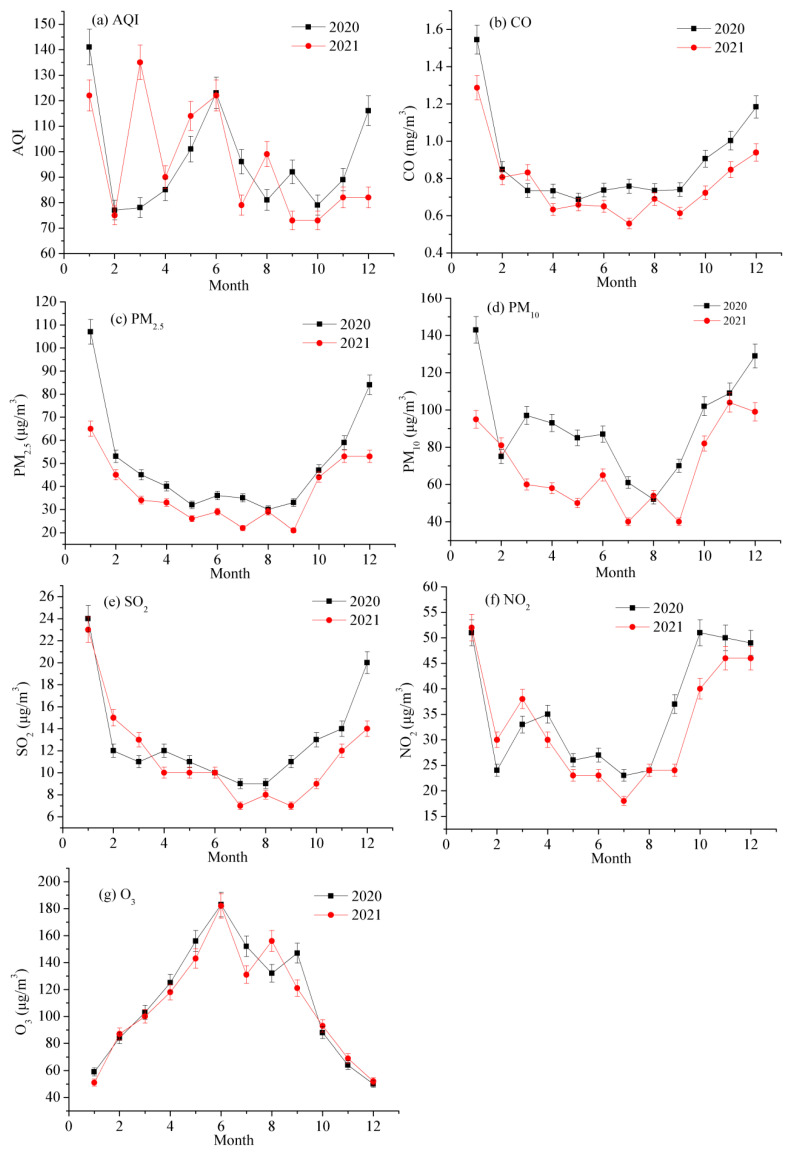
Monthly variations in air quality in Jinan City from 2020 to 2021. (**a**) monthly changes of AQI; (**b**) monthly changes of CO concentrations; (**c**) monthly changes of PM_2.5_ concentrations; (**d**) monthly changes of PM_10_ concentrations; (**e**) monthly changes of SO_2_ concentrations; (**f**) monthly changes of NO_2_ concentrations; (**g**) monthly changes of O_3_ concentrations.

The concentrations of the six air pollutants and AQI in Jinan City have obvious monthly variation characteristics from 2020 to 2021 (Figure 3). The AQI in March, May, and July, 2021, were larger than those in 2020, while those in other months in 2021 were smaller than those in 2020 (Figure 3a). Meanwhile, AQI had its maximum value in January, 2020 and 2021. CO concentration in March, 2021, was higher than that in 2020 and those in other months in 2021 were lower than those in 2020 (Figure 3b). Moreover, the concentrations of CO had maximum values and minimum values in January and August, 2020 and 2021, respectively. PM_2.5_ concentrations in all months in 2021 were smaller than those in 2020 (Figure 3c). Furthermore, the concentrations of PM_2.5_ also had maximum values and minimum values in January and August, 2020 and 2021, respectively. PM_10_ concentrations in February and August, 2021, were larger than those in 2020 and smaller than those in other months (Figure 3d). At the same time, the concentrations of PM_10_ also had maximum values and minimum values in January and August, 2020 and 2021, respectively. SO_2_ concentrations in February and March, 2021, were larger than those in 2020, and SO_2_ concentrations in other months of 2021 were smaller than those in 2020 (Figure 3e). Moreover, the concentrations of SO_2_ also had maximum values and minimum values in January and August, 2020 and 2021, respectively. NO_2_ concentrations from January to March, 2021, were larger than those in 2020, and those in other months of 2021 were smaller than those in 2020 (Figure 3f). Meanwhile, the concentrations of NO_2_ also had maximum values and minimum values in January and August, 2020 and 2021, respectively. O_3_ concentrations in February, October, and December, 2021, were larger than those in 2020, while those in other months of 2021 were smaller than those in 2020 (Figure 3g). However, the concentrations of O_3_ had maximum values and minimum values in June and December, 2020 and 2021, respectively.

**Figure 4:**
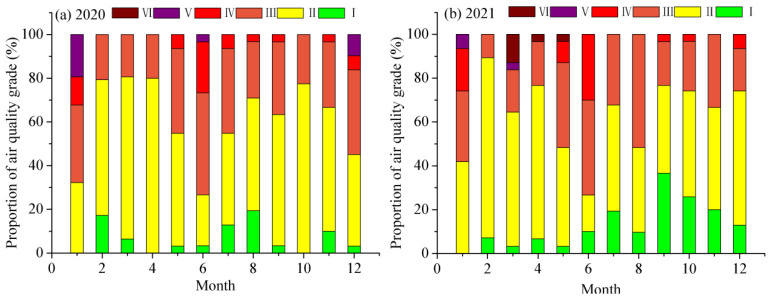
Monthly variations in the proportions of air quality ranks in Jinan City in 2020 and 2021. (**a**) monthly changes of the proportions of AQI ranks in 2020; (**b**) monthly changes of the proportions of AQI ranks in 2021.

We also analyzed the proportions of the AQI ranks in Jinan during January–December in 2020 and 2021, respectively. The total ratios of rank I and rank II in January, February, July, September, and December 2021 were larger than those in 2020, while those in other months were smaller than those in 2020 (Figure 4a,b). It shows that the air quality conspicuously improved in January, February, July, September, and December, 2021, and did not change in June and November, but others remarkably deteriorated.

### 3.7. Hourly Changes in Air Quality in Jinan City

**Figure 5:**
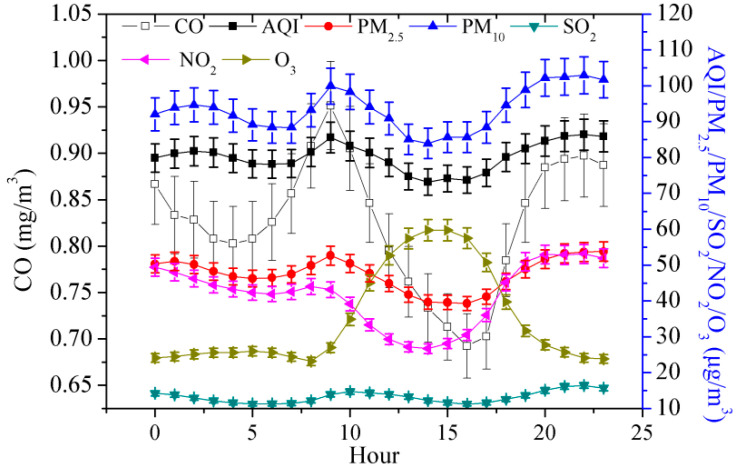
Average hourly variations in air quality in Jinan City.

The concentrations of the six air pollutants and AQI in Jinan City also have obvious hourly variation in their characteristics from 21 November 2022 to 31 December 2022 (Figure 5). The hourly variation trend of air quality (AQI, PM_10_, SO_2_, PM_2.5_, CO, and NO_2_ concentrations) include single valley and double peaks. The first peak appeared at 8–9, the second peak appeared at 22–23, and the lowest appeared at about 16. However, the hourly variation trend of O_3_ concentration is a single peak. The peak appeared at 15.

The correlations between air quality and meteorological factors are shown in Table 7. On the hourly time scale, the sample size (N) was 936. Air quality (AQI, PM_10_, SO_2_, PM_2.5_, CO, and NO_2_ concentrations) was positively correlated with MWS. However, O_3_ concentration was negatively correlated with MWS. MAP was positively correlated with SO_2_ concentration and negatively correlated with AQI, PM_10_, O_3_, PM_2.5_, CO, and NO_2_ concentrations. PR was positively correlated with CO and NO_2_ concentrations and negatively correlated with AQI, PM_10_, SO_2_, PM_2.5_, and O_3_ concentrations. AAT was positively correlated with AQI, PM_10_, O_3_, PM_2.5_, CO, and NO_2_ concentrations and negatively correlated with SO_2_ concentration. ARH was positively correlated with SO_2_ and O_3_ concentrations and negatively correlated with AQI, PM_10_, PM_2.5_, CO, and NO_2_ concentrations.

### 3.8. Comparison with Other Literature

There are many studies on the temporal change characteristics of air quality and its influencing factors on daily, monthly, and annual scales in other cities and regions. Compared to 2014, there were significant decreases of air pollutants in China in 2018, which were about 16% AQI, 20% NO_2_, 25% CO, 20% PM_10_, 52% SO_2_, and 28% PM_2.5_. The continuous improvement of air quality is mainly related with rigorous emission control acts in China, along with the changes in meteorology. In contrast, O_3_ concentration continuously increased during 2014–2018 [51]. PM_10_, PM_2.5_, SO_2_, and CO concentrations in China between 2015 and 2019 decreased, while the O_3_ concentration increased. The increasing rate of O_3_ in ‘2 + 26’ cites was 14 times the global mean. In terms of diurnal variation, CO and NO_2_ concentrations reached their maxima between approximately 8:00 and 9:00 a.m. due to morning rush hour traffic, which was approximately 1 h before the SO_2_ and PMs reached maxima [52].

The average concentrations of five pollutants (PM_10_, PM_2.5_, SO_2_, NO_2_, and CO) decreased by about 15.3%, 19.3%, 29.3%, 9.4%, and 8% from 2015 to 2016 in China. On the contrary, the O_3_ concentration increased by about 4.2% during 2015–2016, which was mainly due to high VOC loading. The concentrations of the five pollutants were the highest and the lowest in winter and summer, respectively. Nevertheless, the O_3_ concentration peaked in summer, followed by ones in spring and autumn and presented the lowest one in winter. The six pollutants exhibited significant diurnal cycle in China. The five pollutants presented the bimodal pattern with two peaks in the morning (9:00–10:00) and at late night (21:00–22:00), respectively. Nevertheless, the O_3_ concentration exhibited the highest value around 15:00. The PM_10_, PM_2.5_, and SO_2_ concentrations were significantly associated with atmosphere temperature, precipitation, and wind speed. The CO and NO_2_ concentrations displayed a significant relationship with atmosphere temperature, while the O_3_ concentration was closely linked to relative humidity and the sunshine duration [53]. Decreases in PM_2.5_, PM_10_, NO_2_, SO_2_, and CO levels were found in about 91%, 92%, 75%, 94%, and 89% of 336 Chinese cities from 2016 to 2020, respectively, while an increase in O_3_ was found in about 87% of 336 Chinese cities [54].

From 2015 to 2019, the annual number of PM_2.5_ (O_3_) pollution days in eastern China decreased (increased) by about 9% (19%). The daily average PM_2.5_ concentrations were positively correlated with the O_3_ concentrations in most regions and seasons in eastern China, and it tended to be more positively correlated as the PM_2.5_ concentration decreased. The temperature was positively correlated with the O_3_ concentration. Under high-temperature conditions, the PM_2.5_ and O_3_ concentrations exhibited a stronger positive correlation. The relative humidity was negatively correlated with the O_3_ concentration and positively correlated with the PM_2.5_ concentration in the North China Plain (NCP), but was negatively correlated with it in the Yangtze River Delta (YRD) and Pearl River Delta (PRD) [55].

In the Beijing–Tianjin–Hebei (BTH) region during 2015–2020, PM_2.5_ pollution decreased significantly, indicating air pollution control policies in China have taken effect. Temperature and precipitation mainly showed negative impacts on PM_2.5_ pollution, while relative humidity, wind speed, and sunshine duration aggravated PM_2.5_ pollution in the BTH [56]. The concentration of air pollutants in the Chengdu–Chongqing urban agglomeration (CCUA) during 2015–2021 has decreased year by year. Except for O_3_, the five air pollutants in autumn and winter were higher than those in summer. The six air pollutants and AQI have dominant periods on multiple time scales. AQI showed positive coherence with PM_2.5_ and PM_10_ on multiple time scales. AQI showed an obvious positive correlation with sunshine hours and temperature and a clear negative correlation with rainfall and humidity [57].

The annual average AQI of all cities in the Yellow River Economic Belt (YREB) decreased from about 107 to 74 during 2014–2019. Annual changes in AQI over the YREB followed a U-shaped pattern, being lower in spring and summer and higher in autumn and winter. The monthly variation cycles of AQI were also distinct over the YREB. Air pollution was most severe from December to February. Air quality was relatively good from June to August. The high AQI of the YREB in winter was associated with residential heating via coal combustion, which is highly polluting. In most northern cities, the extensive emissions in winter, together with weak convection, the low levels of rainfall, and lower vegetation cover, led to the worst air quality among the seasons. Annual wind speed and relative humidity had significant negative effects on the AQI values over the YREB [33].

The concentrations of five air pollutants (SO_2_, NO_2_, CO, PM_10_, and PM_2.5_) decreased from 2006 to 2019, but the O_3_ concentration increased in the Pearl River Delta (PRD). Monthly PM_2.5_ was not significantly correlated with O_3_. However, it had a positive correlation with NO_2_, SO_2_, CO, and PM_10_ concentrations. NO_2_ concentration was significantly correlated with CO concentration. In addition to the significant positive correlation between O_3_ and PM_10_ concentrations, there was also a negative correlation between O_3_ and other pollutant concentrations. In addition to the significant positive correlation between PM_2.5_ concentration and air pressure (AP), PM_2.5_ concentration was also negatively correlated with precipitation (P), relative humidity (RH), sunshine duration (SD), temperature (T), and wind speed (WS). The positive or negative correlations between other pollutants (NO_2_, SO_2_, CO, PM_10_) and meteorological factors were the same as those between PM_2.5_ concentration and meteorological factors. This finding indicated that NO_2_, SO_2_, CO, PM_10_, and PM_2.5_ concentrations were relatively low in places with high P, T, and RH. Moreover, PM_10_, CO, and PM_2.5_ concentrations were negatively correlated with WS, which was mainly because WS was the main driving factor for the diffusion of air pollutants. The higher the wind speed, the more conducive it was for the diffusion and dilution of pollutants. The six air pollutants were negatively correlated with P, indicating that wet scavenging of precipitation was the primary removal method of aerosol particles from the atmosphere. O_3_ concentration had a positive correlation with T and SD. High O_3_ concentrations appeared when T and SD were high. On the monthly time scale, air pollutants had high correlations with RH, AP, P, and T. In different seasons, the correlations among air pollutants and meteorological factors were slightly different [58].

The O_3_ pollution in Beijing, Chengdu, Guangzhou, and Shanghai were more and more serious during 2013–2020. The meteorology is the dominant driver for the O_3_ trend. The variations in meteorology lead to the enhancement of atmospheric oxidation capacity and the acceleration of O_3_ production. Though the NO*_x_*/VOC ratios were obviously decreased from 2013 to 2020, the emission reductions were still not enough to mitigate O_3_ pollution in the four cities [59].

Compared to 2014, BC, PM_2.5_, and PM_10_ concentrations in 2019 in Beijing decreased by about 53.7%, 52.7%, and 46.9%, respectively. However, O_3_ concentration showed an upward trend. There was obvious diurnal variation in CO, NO_2_, SO_2_, and O_3_ concentrations. The CO concentration in summer in 2014–2019 started to rise in the early morning, reached a peak around 9 am, then began to decline, and reached a valley around 4 pm. The NO_2_ concentration showed a similar diurnal variation to CO, reaching a peak at about 4 am and a valley around 3 pm. The SO_2_ concentration reached its lowest value at around 6 am and reached its peak after noon. O_3_ concentration showed a more distinctly unimodal variation, reaching its lowest value around 6 am, reaching its peak at noon under the influence of solar radiation. High temperature, moderate humidity, and sufficient sunlight are conducive to the existence of high concentrations of O_3_ [60].

The annual concentrations of O_3_ in Tianjin showed an overall upward trend during 2014–2019, then decreased significantly during 2020–2021.Temperature was the most important factor affecting O_3_ level, followed by air humidity in O_3_ pollution season. Specifically, in summer, O_3_ pollution frequently exceeded the standard level (>160 µg/m^3^) at combined with a relative humidity of 40–50% and a temperature > 31 °C [61]. The growth of per capita GDP (GDPPC) facilitated the reduction of PM_2.5_ pollution while the increase in the other socioeconomic factors aggravated haze pollution in North China Plain from 2013 to 2017 [62].

The air quality around the world has also changed significantly. The global distribution of average PM_2.5_ concentrations during 1998–2016 shows that PM_2.5_ concentrations were most pronounced in China and India. Values of more than 50% (extreme increase) were widely distributed throughout India and neighboring regions. Sporadic areas of extreme increases were found in South America, Africa, and Asia. In Western Europe and the United States, many areas had decreased PM_2.5_ concentrations in 1998–2016 [63]. PM_2.5_ concentrations in Ulaanbaatar city of Mongolia have been declining since 2018. However, PM_2.5_ from January to March 2020 was about 129, 71, and 33 µg/m^3^, respectively [64]. SO_2_ concentrations in India increased between 1980 and 2010. However, SO_2_ concentration shows a decreasing trend in 2010–2020 [65]. When stratifying the analysis by every 5 years in 10 Japanese cities, average concentrations in each sub-period decreased for SO_2_ and NO_2_ concentrations (about 14–2 ppb and 29–18 ppb, respectively) but increased for Ox concentration (29–39 ppb) during 1977–2015 [66]. Annual mean PM_2.5_ concentrations over North Korea from 2015 to 2018 were about 43.5, 40, 41.1, and 42.7 μg/m^3^. The highest PM_2.5_ concentrations appeared in Pyongyang, with corresponding annual values of 55.7, 50.4, 45.4, and 47.2 μg/m^3^, respectively. The PM_2.5_ concentrations showed declining trends [67]. Both cities of Paris and London had downward trends in background NO_2_ concentrations in 2005–2009 (about −2.1% and −1.4% per year in Paris and London, respectively). In 2010–2016, NO_2_ concentrations in London decreased faster (−2.1% per year) than that in Paris (−1.7% per year). PM_2.5_ concentrations at background locations in Paris decreased at −4.2% per year in 2005–2009 and faster in 2010–2016 at −5.2% per year. London had downward trends in 2005–2016 [68]. Air pollution (NO_2_, PM_10_, and PM_2.5_ concentrations) trends showed an overall decrease in pollution levels in Spain in the 1993–2017 period (2001–2017 for PM_2.5_). In contrast, average ambient O_3_ levels have increased by nearly 10 μg/m^3^ [69]. The average annual population-weighted PM_2.5_ exposure in Europe in 1990 was about 21 μg/m^3^, while in 2019 it was about 34% lower at 14 μg/m^3^ [70]. The annual average PM_2.5_ concentration over North America decreased from about 22 μg/m^3^ in 1981 to 8 μg/m^3^ in 2016, with an overall trend of −0.33 μg/m^3^ per year [71].

The temporal variation in characteristics of air quality in Jinan city are similar to those in other cities, and the impact of meteorological factors on air pollution is also similar. Combined with these findings from previous studies, the air quality improvements in Jinan city should be mainly conducted by rigid air quality control policies and emission reduction measures.

## 4. Conclusions

This study presents the temporal changes (annual, seasonal, monthly, and hourly) in air quality in Jinan City. The annual values of six air pollutants and AQI in Jinan City during 2014–2021 mainly showed a decreasing trend. The seasonal concentrations of five air pollutants (PM_10_, PM_2.5_, NO_2_, SO_2_, and CO) from 2014 to 2021 also decreased gradually, but the O_3_ concentration in winter increased. The concentrations of five air pollutants during 2020–2021 had the highest values and lowest values in January and August, respectively, but the concentrations of O_3_ had the highest values and lowest values in June and December, respectively.

The COVID-19 pandemic has had an unexpected effect on air quality in Jinan City. The AQI in Jinan City during the COVID epoch in 2020 was prominently lower compared with that in the same epoch in 2021. Control measures played a key role in improving AQI in Jinan City. However, the air quality in Jinan City during the post-COVID epoch in 2020 was significantly deteriorated compared with that during the same epoch in 2021.

Air pollutants are highly correlated with MAP, AAT, ARH, SH, MWS, and PR. Moreover, air pollutants are also highly correlated with SDE, NOE, PE, GDPPC, PO, and ECPGDP. The air pollution is the most serious in winter, partly because the weather conditions in winter are more unfavorable to the diffusion of pollutants than in other seasons.

This article focuses on the correlation between air quality, weather elements, and socio-economic factors on an annual scale. We did not analyze the correlation with the time of the year and the weather conditions. In future, the research can be further extended to daily and hourly air pollution and other factors. In addition, other factors, such as cloud, water vapor, wind direction and land use, can be further taken into consideration. More research is needed in the future to confirm the two-way correlations between socio-economic factors and air pollution.

The research results can help us to better understand the influence of meteorological factors and socio-economic factors on air quality. Meanwhile, the research results can provide the required knowledge to optimize the performance of air pollution forecast models and to help management departments to develop scientific control strategies.

With China’s population growth, air pollution is still a public health and economic problem. Policies aimed at reducing air pollution should continue to be vigorously implemented to further reduce the risk. In order to fight the key battle against pollution and cope with climate change in depth, China has put forward the goal of carbon neutrality. China will actively promote clean energy, constantly promote clean production and efficient use of energy resources, and vigorously develop non fossil energy. China will defend the blue sky with a higher standard and strive to build a beautiful China where people and nature coexist harmoniously.

## Figures and Tables

**Table 1:** AQI range and its rank.

Range	Rank	Description
0–50	Ⅰ	Outstanding
51–100	Ⅱ	Well
101–150	Ⅲ	Slight pollution
151–200	Ⅳ	Medium pollution
201–300	Ⅴ	Heavy pollution
>300	Ⅵ	Severe pollution

**Table 2:** Relevant coefficients (R) between air quality, meteorological factors, air pollution emissions, GDPPC, population, and ECPGDP.

Years	MWS	MAP	PR	AAT	ARH	SH	SDE	NOE	PE	GDPPC	PO	ECPGDP
AQI	−0.708	0.302	−0.482	0.177	0.257	0.146	0.904 **	0.907 **	0.832 *	−0.945 **	−0.707 *	0.943 **
PM_2.5_	−0.594	0.416	−0.494	0.140	0.417	0.199	0.940 **	0.953 **	0.847 *	−0.976 **	−0.770 *	0.958 **
PM_10_	−0.674	0.379	−0.516	0.263	0.304	0.230	0.915 **	0.924 **	0.809 *	−0.995 **	−0.809 *	0.984 **
SO_2_	−0.534	0.480	−0.462	0.074	0.295	0.122	0.923 **	0.944 **	0.847 *	−0.900 **	−0.711 *	0.970 **
NO_2_	−0.845 *	0.206	−0.562	0.485	−0.012	0.114	0.818 *	0.758 *	0.732	−0.971 **	−0.824 *	0.910 **
CO	−0.548	0.476	−0.353	0.041	0.566	0.323	0.835 *	0.847 *	0.700	−0.916 **	−0.792 *	0.872 *
O_3_	0.302	−0.463	−0.225	0.337	−0.690	−0.489	−0.668	−0.684	−0.481	0.573	0.640	−0.698
Days	MWS	MAP	PR	AAT	ARH	SH						
AQI	−0.224 **	0.046 *	−0.181 **	−0.075 **	0.039 *	−0.055 **						
PM_2.5_	−0.280 **	0.278 **	−0.141 **	−0.338 **	0.162 **	−0.039 *						
PM_10_	−0.161 **	0.229 **	−0.209 **	−0.269 **	−0.078 **	0.002						
SO_2_	−0.175 **	0.323 **	−0.139 **	−0.383 **	−0.169 **	0.050 *						
NO_2_	−0.461 **	0.490 **	−0.166 **	−0.421 **	−0.056 **	0.039 *						
CO	−0.347 **	0.346 **	−0.087 **	−0.410 **	0.198 **	−0.063 **						
O_3_	0.139 **	−0.697 **	−0.026	0.811 **	−0.116 **	0.012						

** expresses *p* < 0.01. * expresses *p* < 0.05. Years (N = 8), Days (N = 2922).

**Table 3:** R between six air pollutant and AQI values.

Years	AQI	PM_2.5_	PM_10_	SO_2_	NO_2_	CO	O_3_
AQI	1.000	0.977 **	0.970 **	0.940 **	0.925 **	0.911 **	−0.599
PM_2.5_	0.977 **	1.000	0.990 **	0.925 **	0.923 **	0.952 **	−0.646
PM_10_	0.970 **	0.990 **	1.000	0.928 **	0.960 **	0.926 **	−0.584
SO_2_	0.940 **	0.925 **	0.928 **	1.000	0.846 **	0.819 *	−0.530
NO_2_	0.925 **	0.923 **	0.960 **	0.846 **	1.000	0.844 **	−0.455
CO	0.911 **	0.952 **	0.926 **	0.819 *	0.844 **	1.000	−0.806 *
O_3_	−0.599	−0.646	−0.584	−0.530	−0.455	−0.806 *	1.000
Days	AQI	PM_2.5_	PM_10_	SO_2_	NO_2_	CO	O_3_
AQI	1	0.872 **	0.846 **	0.451 **	0.501 **	0.679 **	0.049 **
PM_2.5_	0.872 **	1	0.901 **	0.544 **	0.627 **	0.798 **	−0.291 **
PM_10_	0.846 **	0.901 **	1	0.608 **	0.679 **	0.756 **	−0.233 **
SO_2_	0.451 **	0.544 **	0.608 **	1	0.597 **	0.646 **	−0.314 **
NO_2_	0.501 **	0.627 **	0.679 **	0.597 **	1	0.744 **	−0.426 **
CO	0.679 **	0.798 **	0.756 **	0.646 **	0.744 **	1	−0.421 **
O_3_	0.049 **	−0.291 **	−0.233 **	−0.314 **	−0.426 **	−0.421 **	1
2014	AQI	PM_2.5_	PM_10_	SO_2_	NO_2_	CO	O_3_
AQI	1	0.931 **	0.916 **	0.538 **	0.540 **	0.745 **	−0.168 **
PM_2.5_	0.931 **	1	0.903 **	0.598 **	0.612 **	0.835 **	−0.317 **
PM_10_	0.916 **	0.903 **	1	0.593 **	0.640 **	0.738 **	−0.296 **
SO_2_	0.538 **	0.598 **	0.593 **	1	0.724 **	0.745 **	−0.552 **
NO_2_	0.540 **	0.612 **	0.640 **	0.724 **	1	0.706 **	−0.483 **
CO	0.745 **	0.835 **	0.738 **	0.745 **	0.706 **	1	−0.468 **
O_3_	−0.168 **	−0.317 **	−0.296 **	−0.552 **	−0.483 **	−0.468 **	1
2015	AQI	PM_2.5_	PM_10_	SO_2_	NO_2_	CO	O_3_
AQI	1	0.966 **	0.931 **	0.525 **	0.671 **	0.821 **	−0.207 **
PM_2.5_	0.966 **	1	0.899 **	0.530 **	0.694 **	0.852 **	−0.295 **
PM_10_	0.931 **	0.899 **	1	0.598 **	0.703 **	0.780 **	−0.220 **
SO_2_	0.525 **	0.530 **	0.598 **	1	0.741 **	0.680 **	−0.523 **
NO_2_	0.671 **	0.694 **	0.703 **	0.741 **	1	0.807 **	−0.480 **
CO	0.821 **	0.852 **	0.780 **	0.680 **	0.807 **	1	−0.519 **
O_3_	−0.207 **	−0.295 **	−0.220 **	−0.523 **	−0.480 **	−0.519 **	1
2016	AQI	PM_2.5_	PM_10_	SO_2_	NO_2_	CO	O_3_
AQI	1	0.926 **	0.905 **	0.564 **	0.633 **	0.802 **	−0.096
PM_2.5_	0.926 **	1	0.900 **	0.624 **	0.709 **	0.878 **	−0.318 **
PM_10_	0.905 **	0.900 **	1	0.586 **	0.677 **	0.755 **	−0.210 **
SO_2_	0.564 **	0.624 **	0.586 **	1	0.617 **	0.639 **	−0.400 **
NO_2_	0.633 **	0.709 **	0.677 **	0.617 **	1	0.770 **	−0.407 **
CO	0.802 **	0.878 **	0.755 **	0.639 **	0.770 **	1	−0.432 **
O_3_	−0.096	−0.318 **	−0.210 **	−0.400 **	−0.407 **	−0.432 **	1
2017	AQI	PM_2.5_	PM_10_	SO_2_	NO_2_	CO	O_3_
AQI	1	0.871 **	0.853 **	0.472 **	0.470 **	0.725 **	0.059
PM_2.5_	0.871 **	1	0.921 **	0.606 **	0.629 **	0.861 **	−0.311 **
PM_10_	0.853 **	0.921 **	1	0.582 **	0.627 **	0.785 **	−0.260 **
SO_2_	0.472 **	0.606 **	0.582 **	1	0.570 **	0.625 **	−0.385 **
NO_2_	0.470 **	0.629 **	0.627 **	0.570 **	1	0.711 **	−0.451 **
CO	0.725 **	0.861 **	0.785 **	0.625 **	0.711 **	1	−0.439 **
O_3_	0.059	−0.311 **	−0.260 **	−0.385 **	−0.451 **	−0.439 **	1
2018	AQI	PM_2.5_	PM_10_	SO_2_	NO_2_	CO	O_3_
AQI	1	0.717 **	0.737 **	0.290 **	0.329 **	0.527 **	0.285 **
PM_2.5_	0.717 **	1	0.874 **	0.557 **	0.590 **	0.845 **	−0.317 **
PM_10_	0.737 **	0.874 **	1	0.545 **	0.592 **	0.727 **	−0.252 **
SO_2_	0.290 **	0.557 **	0.545 **	1	0.501 **	0.716 **	−0.397 **
NO_2_	0.329 **	0.590 **	0.592 **	0.501 **	1	0.714 **	−0.449 **
CO	0.527 **	0.845 **	0.727 **	0.716 **	0.714 **	1	−0.442 **
O_3_	0.285 **	−0.317 **	−0.252 **	−0.397 **	−0.449 **	−0.442 **	1
2019	AQI	PM_2.5_	PM_10_	SO_2_	NO_2_	CO	O_3_
AQI	1	0.723 **	0.693 **	0.382 **	0.380 **	0.583 **	0.259 **
PM_2.5_	0.723 **	1	0.866 **	0.564 **	0.685 **	0.848 **	−0.362 **
PM_10_	0.693 **	0.866 **	1	0.602 **	0.685 **	0.701 **	−0.290 **
SO_2_	0.382 **	0.564 **	0.602 **	1	0.719 **	0.636 **	−0.324 **
NO_2_	0.380 **	0.685 **	0.685 **	0.719 **	1	0.747 **	−0.442 **
CO	0.583 **	0.848 **	0.701 **	0.636 **	0.747 **	1	−0.424 **
O_3_	0.259 **	−0.362 **	−0.290 **	−0.324 **	−0.442 **	−0.424 **	1
2020	AQI	PM_2.5_	PM_10_	SO_2_	NO_2_	CO	O_3_
AQI	1	0.725 **	0.744 **	0.462 **	0.374 **	0.617 **	0.276 **
PM_2.5_	0.725 **	1	0.851 **	0.635 **	0.620 **	0.877 **	−0.347 **
PM_10_	0.744 **	0.851 **	1	0.630 **	0.664 **	0.728 **	−0.164 **
SO_2_	0.462 **	0.635 **	0.630 **	1	0.636 **	0.712 **	−0.326 **
NO_2_	0.374 **	0.620 **	0.664 **	0.636 **	1	0.737 **	−0.407 **
CO	0.617 **	0.877 **	0.728 **	0.712 **	0.737 **	1	−0.403 **
O_3_	0.276 **	−0.347 **	−0.164 **	−0.326 **	−0.407 **	−0.403 **	1
2021	AQI	PM_2.5_	PM_10_	SO_2_	NO_2_	CO	O_3_
AQI	1	0.885 **	0.857 **	0.225 **	0.214 **	0.334 **	0.228 **
PM_2.5_	0.885 **	1	0.944 **	0.286 **	0.354 **	0.417 **	−0.159 **
PM_10_	0.857 **	0.944 **	1	0.452 **	0.510 **	0.538 **	−0.177 **
SO_2_	0.225 **	0.286 **	0.452 **	1	0.705 **	0.762 **	−0.367 **
NO_2_	0.214 **	0.354 **	0.510 **	0.705 **	1	0.838 **	−0.456 **
CO	0.334 **	0.417 **	0.538 **	0.762 **	0.838 **	1	−0.358 **
O_3_	0.228 **	−0.159 **	−0.177 **	−0.367 **	−0.456 **	−0.358 **	1

** expresses *p* < 0.01. * expresses *p* < 0.05. Years (N = 8), Days (N = 2922).

**Table 4:** The ratios of AQI rank from 2014 to 2021 in Jinan City (%).

Season	Year	Ⅰ	Ⅱ	Ⅲ	Ⅳ	Ⅴ	Ⅵ
Spring	2014	0.0	23.9	51.1	16.3	7.6	1.1
	2021	4.4	58.8	26.0	3.2	1.1	6.5
Summer	2014	0.0	25.0	59.8	15.2	0.0	0.0
	2021	13.0	34.6	42.4	10.0	0.0	0.0
Autumn	2014	0.0	33.0	51.6	8.8	5.5	1.1
	2021	27.5	45.0	25.3	2.2	0.0	0.0
Winter	2014	0.0	22.7	36.4	17.0	20.5	3.4
	2021	6.7	61.8	20.8	8.6	2.2	0.0

**Table 5:** Average values of air quality during the COVID epoch in 2020 (and the same epoch in 2021) (µg m^–3^(CO (mg m^–3^))).

Epoch I	AQI	PM_2.5_	PM_10_	SO_2_	NO_2_	CO	O_3_
2020	80.0	46.0	88.3	11.7	30.7	0.77	104.0
2021	100.0	37.3	66.3	12.7	32.7	0.76	101.7

**Table 6:** Average values of air quality during the post-COVID epoch in 2020 (and the same epoch in 2021) (µg m^–3^(CO (mg m^–3^))).

Epoch II	AQI	PM_2.5_	PM_10_	SO_2_	NO_2_	CO	O_3_
2020	94.4	38.9	80.9	11.0	34.0	0.80	131.7
2021	90.5	34.6	66.8	9.6	30.5	0.71	118.4

**Table 7:** Correlations between air quality and meteorological elements.

Hours	MWS	MAP	PR	AAT	ARH
AQI	−0.109 **	−0.052	−0.038	0.059	0.181 **
PM_2.5_	−0.173 **	−0.058	−0.016	0.141 **	0.307 **
PM_10_	−0.076 *	−0.103 **	−0.025	0.050	0.121 **
SO_2_	−0.343 **	0.165 **	−0.081 *	−0.294 **	−0.175 **
NO_2_	−0.239 **	−0.075 *	0.011	0.076 *	0.231 **
CO	−0.156 **	−0.196 **	0.020	0.270 **	0.404 **
O_3_	0.097 **	−0.043	−0.099 **	0.025	−0.100 **

** expresses *p* < 0.01. * expresses *p* < 0.05. (N = 936).

## Data Availability

Not applicable.
